# Non operative management of proximal posterior gastric injury in a trauma patient: A case report

**DOI:** 10.1016/j.ijscr.2020.11.031

**Published:** 2020-11-25

**Authors:** Norah Alsubaie, Bushr Mrad, Abdullah Albdah, Nadia Aljomah, Thamer Nouh

**Affiliations:** a Trauma and Acute Care Surgery Unit, Department of Surgery, College of Medicine, King Saud University, Riyadh, Saudi Arabia; b General Surgery Department, Surgery Department, Prince Mohmammed Bin Abdulaziz Hospital, Saudi Arabia; c Trauma and Acute Care Surgery Unit, Department of Surgery, King Saud University Medical City, Riyadh, Saudi Arabia

**Keywords:** Proximal posterior gastric injury, Blunt abdominal trauma, Nonoperative management

## Abstract

**Introduction:**

Proximal postierior gastric injuries are challenging and are often overlooked and diagnosed late. We present a case of traumatic proximal posterior gastric injury.

**Presentation of case:**

A 34-year-old male patient presented with traumatic proximal posterior gastric injury secondary to falling from a height. Nonoperative treatment was achieved by placing a gastric stent and wide drainage that resulted in complete healing of the perforation within a period of 8 weeks.

**Discussion:**

There are no standard treatment guidelines for managing gastric leaks, except for surgical repair, in trauma patients. Management with a gastric stent has been proven effective in gastric leaks resulting from bariatric surgeries and surgical interventions for gastric cancer. This approach was followed in the management of our patient and showed promising results.

**Conclusion:**

Nonoperative management with an endoscopic stent can be considered a treatment option for leaks resulting from traumatic gastric injury.

## Highlights

• How to navigate the traumatic gastric injury diagnosis dilemma pre operativelly.
• Currently, a gastric leak after surgical repair is associated with significant and prolonged morbidity and mortality, remaining one of the most feared complications with no standard treatment guidelines.
• Managing traumatic GE junction injury with endoscopic stenting resulted in many advantageous including resume oral intake earlier, shorter hospital stay and less morbidities.

## 1 Introduction

proximal posterior gastric injury resulting from blunt trauma have rarely been described in the medical literature [1,5]. They are challenging and often diagnosed late. We present a case of traumatic proximal posterior gastric injury in a 34-year-old man. Most trauma patients are treated with adequate surgical repair or re-exploration if surgery fails. We took a unique management approach by performing endoscopic stenting to manage a gastric leak, which is usually used in post-gastrectomy procedures. Although this approach is not commonly followed in trauma patients, it succeeded, and we have provided further details of management below. This case was reported in compliance with the SCARE guidelines [6].

## 2 Case report

A 34-year-old male patient presented to our center following a fall from a height. The patient had no history of medical illness, family history, or history of drug intake. The patient was resuscitated and managed according to the advanced trauma life support protocol, and he was found to be a transient responder (bleeding 20–40%).

Focused assessment sonography in trauma was positive for fluid in Morrison's pouch. Computed tomography (CT) showed multiple bilateral nondisplaced rib fractures, comminuted scapular fractures, and bilateral lung contusions. Furthermore, abdominal injuries were found, such as grade 4 liver laceration, left renal artery injury resulting in left kidney devascularization, and a right adrenal hematoma. Although initially responsive, following a CT scan, the patient became vitally unstable. Therefore, resuscitation was started, and a plan to take him to the operative room was made.

Intraoperatively, the liver was found to be the primary site of hemorrhage, which was controlled with packing. The lesser sac was opened, and no blood or bile was found, and postrior pastric wall was inspected with no injuries,

Additionally, a serosal tear was found in the transverse colon, which was primarily repaired with continuous PDS suture. Packing with temporary closure of the abdomen was performed (damage control laparotomy), and the patient was sent to the intensive care unit (ICU).

Forty-eight hours later, the patient was taken to the operating room (OR) for a second look. Exploration was uneventful, and the abdomen was successfully closed.

Despite an initial uneventful postoperative recovery, 2 weeks after surgery, the patient’s condition deteriorated. Abdominal examination suggested the presence of gas coming from the upper part of the laparotomy wound, and a diagnosis of septic shock was made. Following resuscitation, the patient underwent an emergency exploratory laparotomy, which was performed by an on-call trained trauma consultant.

**Fig. 1:**
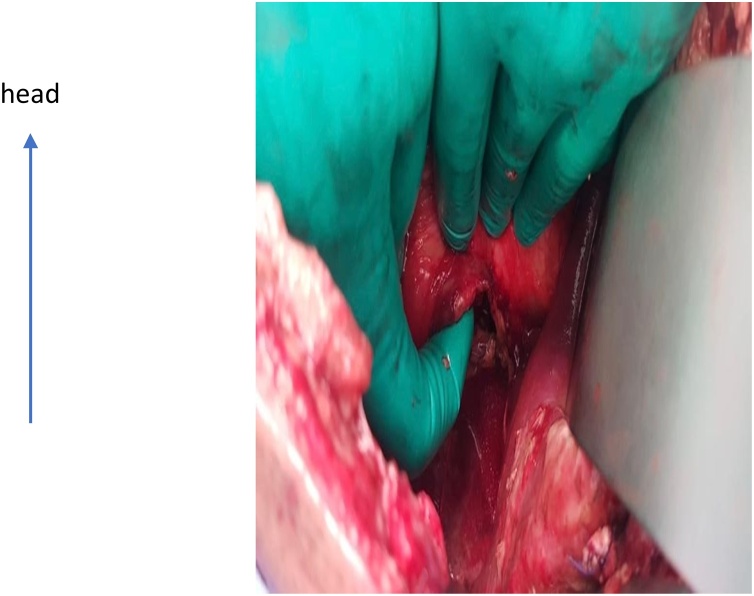
Posterior injury of the gastro-esophageal junction. We ensure that the figures do not contain confidential patient details.

Upon exploration, a 2-cm long, full-thickness posterior gastric injury was found at the level of the cardia just below the GE junction. The necrotic and ischemic margins were debrided resulting in a larger defect that extended up to the GE junction (Fig. 1). Despite the difficulty in gaining good exposure of the injury, primary two-layer closure of the gastric rupture was performed with continuous 2.0 PDS sutures (Z775D) and reinforced with Vicryl 2.0 (J589H) interrupted sutures. No other injuries were found. The patient was sent to the surgical ICU with an open abdomen.

After 48 hrs of observation, the patient was returned to the OR. A jejunostomy feeding tube was inserted, and the abdomen was closed using an abdominal wall closure system device (ABRA; CWK08, Southmedic Inc. 50 alliance Blvd, Barrie, Ontario, Canada).

During the third exploration, the gastric injury that was previously repaired showed a persistent leak. A trial of nonoperative treatment with a Niti-S esophageal BETA-2 covered stent (size 28 mm × 180 mm, made in Korea, LBN 00-01) and wide drainage were initiated through gastroscopy.

**Fig. 2:**
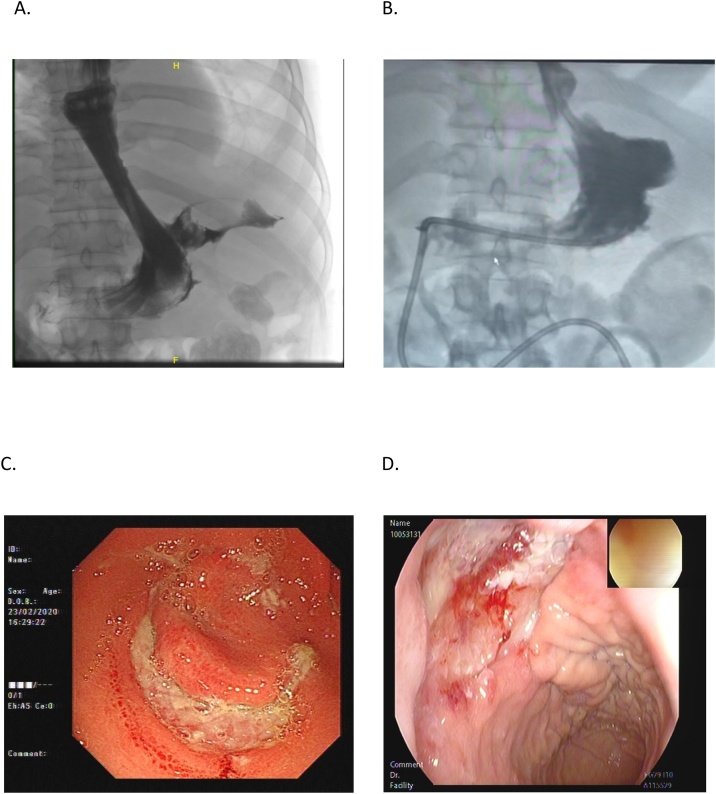
(A) Fluoroscopy showing para-stent leak. (B) Follow-up fluoroscopy with the perforation completely healed. (C), (D) A gastric ulcer that was found at the level of the pylorus upon endoscopy. We ensure that the figures do not contain confidential patient details.

Five weeks later, a fluoroscopy study showed a para-stent leak at the proximal end (Fig. 2). Later on, upon endoscopy for the replacement of the stent, the previously noted perforation decreased in size. However, a gastric ulcer was found at the level of the pylorus (the distal end of the stent) (Fig. 2), so re-stenting was deferred. Conservative management was planned in the form of jejunostomy tube feeding and 40 mg proton pump inhibitor was given for 2 weeks. Furthermore, endoscopic stenting was performed. The patient tolerated the consequences of the above management option with complete satisfaction.

## 3 Discussion

The literature has few reviews of traumatic proximal posterior gastric injury The unique location of the upper stomach and the GE junction, which are protected by the thoracic cage, makes those sites less susceptible to injury. Furthermore, the mobility of the stomach and the thickness of the gastric wall are protective structural features [1].

It was found that blunt gastric injuries are usually associated with other intra- and extra-abdominal injuries, most commonly splenic injuries, which are found in 27% of cases. Isolated injuries are less common, and this can be explained by the mechanism of injury itself, which is most commonly associated with vehicle accidents [2,3].

The main dilemma in cases of traumatic gastric injury is the difficulty in making a preoperative diagnosis [3]. Abdominal pain and peritoneal signs are the most frequent clinical findings. However, these findings are not specific or common among trauma victims. Furthermore, physical examination may be misleading in intoxicated patients or those with distracting injuries to the head, spinal cord, chest, or extremities.

At present, CT is advocated when there is diagnostic doubt in the setting of hemodynamic stability [1,5]. The high risk of mortality is a major concern, making early recognition and management crucial. The management approach varies based on the nature of the injury, the extent of the laceration, and the presence of gastric tissue loss and devascularization [1].

The mainstay of treatment of full-thickness gastric lacerations resulting from BAT is adequate surgical repair. There are no standard treatment guidelines for the treatment of gastric leak after surgical repair for gastric injury in trauma patients, and re-exploration is the most commonly used option.

Nonoperative management with gastric stent has been used and proven effective in gastric leaks resulting from bariatric surgeries and surgical interventions of gastric cancer. In fact, it is associated with a closure rate of 92%–96% [4,7,8]. This approach was sought in our patient and showed promising results. Complete resolution of the leak occurred despite the traumatic nature of the injury.

Compared to reoperation, a gastric stent is a less invasive procedure, associated with decreased morbidity, mortality, and hospital stay. Additionally, stenting is advantageous because it allows patients to resume oral intake earlier [7,8]. However, our patient had multiple complications, including distal ulceration, migration, and para-stent leak. Choi et al. reported complications such as bleeding, stent fracture, stent impaction, and aorto-esophageal fistula. The optimal timing of stent removal is important as late removal increases the complication rate [7,8]. If the above less invasive management is followed in the future, it will provide a more convenient treatment option with less pain, less operative stay, and faster recovery.

## 4 Conclusion

Nonoperative management with an endoscopic stent can be considered a treatment option for leaks resulting from traumatic gastric injury.

## Declaration of Competing Interest

The authors report no declarations of interest.

## Funding

None.

## Ethical approval

This case report was approved by the IRB committee of king Saud university medical city, Vice Rectorate for Graduate Studies & Scientific Research.

## Consent

Written informed consent was obtained from the patient for publication of this case report and accompanying images. A copy of the written consent is available for review by the Editor-in-Chief of this journal on request.

## Author contribution

All authors participated in writing the paper and revising it before submission.

## Registration of research studies

Not applicable.

## Guarantor

Dr Norah Alsubaie.

## Provenance and peer review

Not commissioned, externally peer-reviewed.

## References

[1] Aboobakar M.R., Singh J.P., Maharaj K., Mewa Kinoo S., Singh B. (2017). Gastric perforation following blunt abdominal trauma. Trauma Case Rep..

[2] Hota P.K., Babu M., Satyam G., Praveen A.C. (2014). Traumatic gastric rupture following blunt trauma abdomen: a case series. Bali Med. J..

[3] Bruscagin V., Coimbra R., Rasslan S., Abrantes W.L., Souza H.P., Neto G., Dalcin R.R., Drumond D.A., Ribas J.R. (2001). Blunt gastric injury: a multicentre experience. Injury.

[4] Choi C.W., Kang D.H., Kim H.W., Park S.B., Kim S.J., Hwang S.H., Lee S.H. (2017). Full covered self-expandable metal stents for the treatment of anastomotic leak using a silk thread. Medicine.

[5] Solazzo A., Lassandro G., Lassandro F. (2017). Gastric blunt traumatic injuries: a computed tomography grading classification. World J. Radiol..

[6] Agha R.A., Borrelli M.R., Farwana R., Koshy K., Fowler A.J., Orgill D.P., for the SCARE Group (2018). The SCARE 2018 statement: updating consensus surgical CAse REport (SCARE) guidelines. Int. J. Surg..

[7] Kim J., Azagury D., Eisenberg D., DeMaria E., Campos G.M., American Society for Metabolic and Bariatric Surgery Clinical Issues Committee (2015). ASMBS position statement on prevention, detection, and treatment of gastrointestinal leak after gastric bypass and sleeve gastrectomy, including the roles of imaging, surgical exploration, and nonoperative management. Surg. Obes. Relat. Dis..

[8] Rogalski P., Swidnicka-Siergiejko A., Wasielica-Berger J., Zienkiewicz D., Wieckowska B., Wroblewski E., Baniukiewicz A., Rogalska-Plonska M., Siergiejko G., Dabrowski A., Daniluk J. (2020). Endoscopic management of leaks and fistulas after bariatric surgery: a systematic review and meta-analysis. Surg. Endosc..

